# Targeted disruption of Noc4l leads to preimplantation embryonic lethality in mice

**DOI:** 10.1007/s13238-016-0335-9

**Published:** 2016-12-24

**Authors:** Yongli Qin, Haifeng Li, Lina Jia, Jinghua Yan, George Fu Gao, Xiangdong Li

**Affiliations:** 1 State Key Laboratory of the Agro-Biotechnology, College of Biological Sciences, China Agricultural University, Beijing, 100193 China; 2 CAS Key Laboratory of Pathogenic Microbiology and Immunology, Institute of Microbiology, Chinese Academy of Sciences, Beijing, 100101 China

**Dear Editor,**

Human Nucleolar complex associated 4 homolog (NOC4L, GenBank accession number NM_024078) is the human ortholog of yeast Noc4p. However, with few functional studies evaluated, the physiological contributions of NOC4L have yet to be defined. The Noc4p gene in yeast encodes a 63-kilodalton (KDa) protein that forms a stable heterodimer with Nop14p, which mediates the maturation and nuclear export of 40S ribosomal subunits (Dlakic and Tollervey, 2004; Kuhn et al., 2009; Liu and Thiele, 2001; Milkereit et al., 2003). In yeast, Noc4p temperature-sensitive mutants grow poorly at 37°C (Milkereit et al., 2003), suggesting that Noc4p is important for the growth of yeast. The human *NOC4L* encodes an approximately 58-KDa protein comprising 516 amino acids. Similar to Noc4p, NOC4L contains a Noc domain (residue 416–460 aa) that is composed of a highly conserved stretch of 45 amino acids at its C-terminus (Kuhn et al., 2009; Milkereit et al., 2001; Milkereit et al., 2003).

In order to investigate the function of NOC4L, we first compared the amino acid sequences of NOC4L from different species. The phylogenetic analysis indicated that putative homologues of Noc4p are present in many eukaryotic species and are conserved across multiple species (Fig. S1). The human NOC4L protein is 79.1% homologous with mouse Noc4l and 36.1% homologous with yeast Noc4p. Important clues about gene function are closely related to the tissue-specific expression pattern of the corresponding mRNA. Next, we evaluated the expression pattern of the Noc4l gene in adult mouse tissues using RT-qPCR. Tissues derived from the heart, brain, liver, lung, kidney, small intestine, colon, muscle, epididymal fat (EpiWAT), brown adipose tissue (BAT), lymphoid organs (*i.e.,* spleen, thymus, lymphaden) and testes were used for this analysis. The result showed that Noc4l was ubiquitously expressed in all tissues, particularly in highly proliferative tissues such as testes and lymphoid organs (Fig. 1A). The expression pattern of Noc4l mRNA is very similar to the expression of 18S rRNA processing genes which are ubiquitously expressed because ribosome biogenesis is an essential process. We further examined the expression profile of Noc4l in the preimplantation embryos, because many genes associated with ribosome biogenesis play a key role in embryonic development such as RBM19, fibrillarin or WDR36 (Gallenberger et al., 2011; Newton et al., 2003; Zhang et al., 2008). Oocytes, fertilized eggs, 2-cell embryos, morulae and blastocysts were isolated from the timed pregnant mice, and embryos of the same stage were pooled together into one sample. RT-qPCR revealed that the expression level of Noc4l mRNA differed among embryos at different stages of development (Fig. 1B). It is noteworthy that Noc4l mRNA was robust in oocytes and one-cell embryos as well as morula stage (Fig. 1B). Additionally, we analyzed Noc4l protein expression by immunofluorescence assay. We found that Noc4l is mainly localized in the cytoplasmic granules and Noc4l protein was first detected at the 8-cell stage embryos and also consistently observed at the morula and blastocyst stage embryos (Fig. 1C). This suggests that Noc4l may be activated for translation at a specific time in the gamete to embryo transition because of timely translation during the mouse oocyte-to-embryo transition (Oh et al., 2000). Notably, genes involved in ribosome biogenesis are transiently and preferentially expressed in 8-cell embryos (Zeng et al., 2004). The results presented in Fig. 1C demonstrate that Noc4l protein expression significantly increased at the 8-cell embryos stage which further implies that Noc4l may play a key role in the biogenesis of 40S ribosomal subunit like yeast Noc4p.

**Figure 1:**
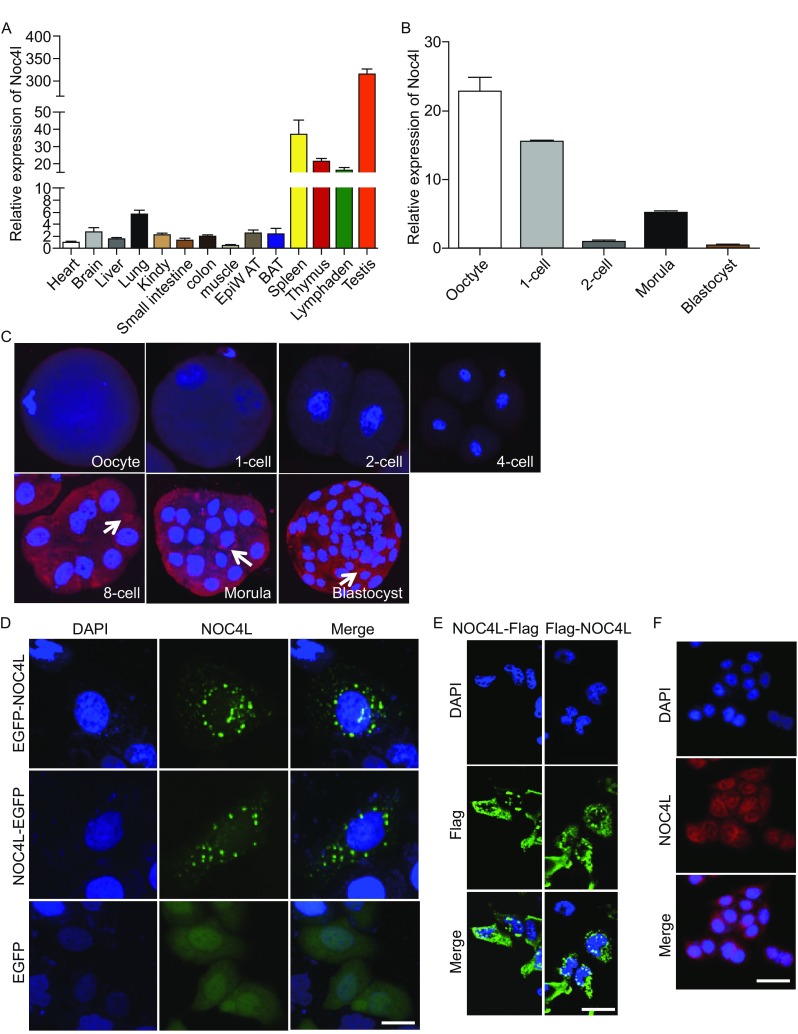
**The expression pattern of Noc4l in mouse tissues and embryos and intracellular localization of NOC4L in Hela cells**. (A) Noc4l mRNA expression profiles in various tissues of adult mice. The mean value obtained from heart tissue was set as 1. GAPDH was used as a reference gene. *n* = 3 mice. (B) qPCR analysis of Noc4l mRNA in mouse oocytes and preimplantation embryos. GAPDH was used as an internal control. The results are presented as the mean ± SEM. (C) Immunofluorescence analysis of Noc4l protein from the oocyte to the blastocyst stage. The expression of Noc4l (red) was observed in 8-cell stage, morulae and blastocysts as white arrows indicated. The nuclei were stained with DAPI. (D) Either an EGFP-NOC4L or NOC4L-EGFP protein expression vectors were transiently transfected into Hela cells. One day after transfection, the EGFP signals were detected with a fluorescence microscope. Empty vector was transfected as a control. Bar, 5 μm. (E) Flag-NOC4L or NOC4L-Flag-expression vectors were transiently transfected into Hela cells. After 24 h transfection, the Flag signals were detected with the primary Flag antibody followed by FITC-labeled secondary antibody. Bar, 20 μm. (F) NOC4L localization in Hela cells was detected with the primary antibody against NOC4L followed by Alexa Fluor 594-conjugated secondary antibody. Bar, 20 μm

The results (Fig. 1C) indicated that Noc4l is mainly localized in the cytoplasmic granules. Therefore, we next determined the subcellular localization of NOC4L. EGFP was fused to either the N- or C-terminal of the NOC4L to construct the EGFP-NOC4L or NOC4L-EGFP expression plasmids which were transfected into Hela cells, respectively. The signals were detected by fluorescence microscopy. Both EGFP-NOC4L and NOC4L-EGFP localized predominantly in the cytoplasm, especially perinuclear membrane granule-like organelles, whereas EGFP alone as a control was detected throughout the cell (Fig. 1D). Additionally, some faint EGFP signals were seen in the nucleus. In an independent approach, we overexpressed recombinant Flag-tagged NOC4L in Hela cells, including Flag-NOC4L and NOC4L-Flag in which the Flag-tag recombined with the 5′-end and 3′-end of NOC4L, respectively. Immunocytochemistry with antibody against the Flag-tag predominantly visualized strong immunoreactivity in the cytoplasmic granules of cells (Fig.1E). In addition, we also directly observed the localization of NOC4L in Hela cells with the antibody against NOC4L by immunofluorescence assay (Fig.1F). The localization of NOC4L is different from Noc4p which is reported to localize to the nucleolus (Milkereit et al., 2003). Moreover, we didn’t predict the nuclear localization signal (NLS) of NOC4L according to NLStradamus progress (Ba et al., 2009). These results indicated that NOC4L may be in the nucleolus to function in 18S rRNA processing like yeast Noc4p by forming complex with binding partners that carry the NLS. Additionally, NOC4L localization in the cytoplasm implies that NOC4L has other uncharacterized functions, as many nucleolar proteins have extraribosomal function and these proteins also localize in the cytoplasm (Boisvert et al., 2007; Warner and McIntosh, 2009).

To explore the functional roles of Noc4l in vivo, we generated a Noc4l conditional knockout mouse model (Noc4l^flox/flox^) with loxP sites flanking exon 3 of the Noc4l gene (Fig. S2A and S2B). Noc4l^flox/flox^ mice were crossed with EIIa-Cre transgenic mice to facilitate the deletion of exon 3 of Noc4l and generate heterozygous Noc4l mice (Noc4l^+/-^) (Fig. S2C). This targeted disruption results in a frame shift mutation that leads to the early termination of Noc4l protein translation. Mice heterozygous for the Noc4l mutation were viable, fertile and exhibited no apparent abnormalities when observed over a long period of time. However, no homozygous Noc4l^-/-^ mice were recovered from intercrossed Noc4l^+/-^ mice. Genotyping of the offspring derived from the intercrosses of the Noc4l heterozygous female and male mice revealed that 66 out of 204 pups were homozygous for wild-type Noc4l, while the remaining 138 were heterozygous for the Noc4l mutation (Table S1). No homozygous offspring were identified. The ratio of the heterozygous and the wild-type offspring was approximately 2:1, consistent with the expected Mendelian frequency. This result suggested that embryonic lethality resulted from the lack of functional Noc4l alleles. To determine the exact time of death of the homozygous mutant embryos, we isolated and examined embryos generated from the Noc4l^+/-^ intercrosses at different days of gestation. A total of 120 embryos at stages E8.5 to E16.5 were collected and analyzed. No homozygous mutant embryos were identified among the embryos collected at these stages (Table S1), indicating that Noc4l is indispensable for the development of either preimplantation or peri-implantation embryos.

Next, we collected 114 2-cell embryos from the Noc4l heterozygotes intercrosses and directly genotyped them. Among the 114 2-cell embryos, 32 embryos (28.1%) had the wild-type genotype, while 57 (50%) embryos were heterozygous mutants (Fig. 2A and Table S1). The remaining 25 embryos were Noc4l^-/-^ mutants, accounting for 21.9% of the embryos evaluated (Fig. 2A and Table S1). These results demonstrated that Noc4l^-/-^ mutants were present at the 2-cell embryonic developmental stage at the expected Mendelian ratio. To observe the developmental potential of the preimplantation embryos derived from the heterozygous intercrosses, we collected additional 55 2-cell embryos and placed them into individual wells of 96-well plates for observation and imaging over a 3-day incubation period in vitro. Subsequently, the 55 embryos were lysed and genotyped, respectively (Table S1). One day after being placed in culture (E2.5), all of the embryos had developed to the 8- to 16-cell stages (Fig. 2B). At E3.5, the wild-type and Noc4l heterozygotes continued to develop into blastocysts with a blastocoel and an inner cell mass. However, although Noc4l^-/-^ embryos were able to compact at the 8- to 16-cell stages to form the morula, they ceased to further develop into blastocysts. At E4.5, the homozygous embryos had completely deteriorated. These embryos lacked the inner cavity or blastocoel observed in blastocysts, and the embryonic cells became detached from the zona pellucida and aggregated into a cluster (Fig. 2B). By contrast, the wild-type and heterozygous embryos appeared prepared to hatch. The appearances of the 55 embryos observed are summarized in Fig. 2B. In summary, the analysis of cultured embryos confirmed that the loss of Noc4l causes lethality during the morula stage of embryonic development and prevents the formation of blastocysts.

**Figure 2:**
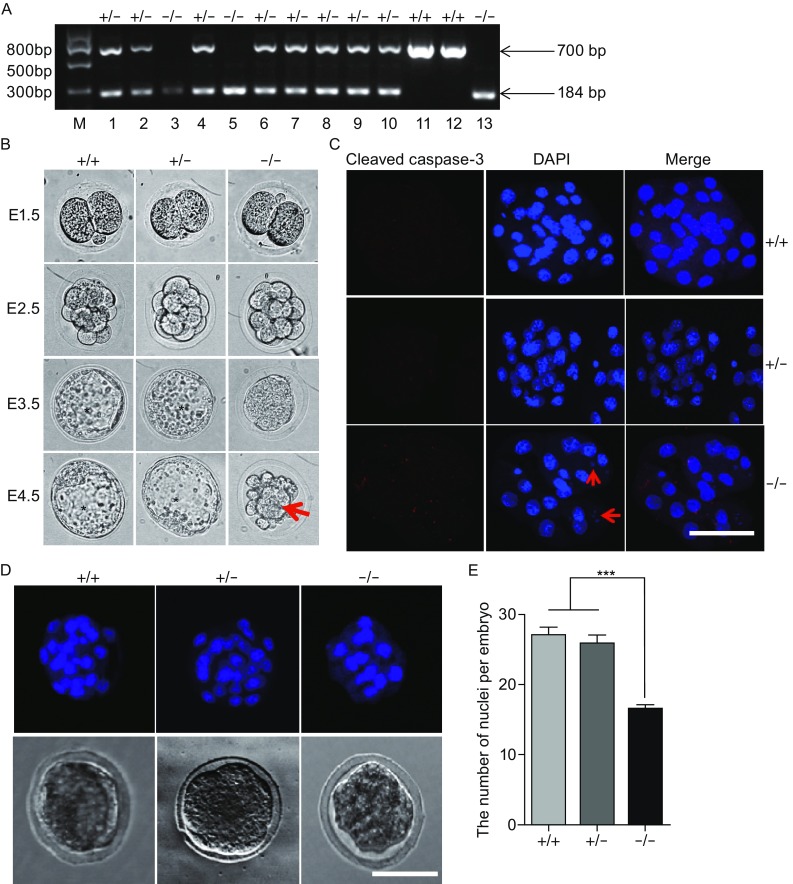
**Noc4l deficiency results in early embryonic lethality**. (A) Two-cell embryos genotyped using nested PCR. PCR products (1–13 of 114) from 2-cell embryos are shown. Amplification of the wild-type allele and the targeted allele generated 700-bp and 184-bp PCR products, respectively. M, marker; +/+, wild-type; +/−, heterozygote; −/−, homozygote. (B) Morphological analysis of preimplantation embryos. Embryos were collected at E1.5 (2-cell stage) from Noc4l^+/−^ heterozygote intercrosses and cultured in vitro for three days. These embryos were imaged at E1.5, 2.5, 3.5 and 4.5. Wild-type (+/+) and heterozygous (+/−) Noc4l mutant embryos developed normally and reached the early blastocyst stage at E3.5. In contrast, homozygous (−/−) Noc4l-deficient embryos did not reach the blastocyst stage and deteriorated by E4.5. In homozygous Noc4l-deficient embryos, cells became detached from the zona pellucida and aggregated together to form a cluster (Red arrow). Asterisks indicate the blastocoel. (C) Immunofluorescence analysis of morulae for the apoptotic marker activated caspase-3. Embryos isolated at E2.5 were cultured for 12 h in KSOM. Activated caspase-3 was detected by immunofluorescence staining for cleaved caspase-3 (red). Nuclear DNA was stained with DAPI. The red arrows indicate fragmentation of chromatin. Bar, 30 μm. (D) DAPI nuclear staining of morula embryos. The DAPI images and brightfield images are presented in the upper and lower panel, respectively. Bar, 50 μm. (E) The number of nuclei per embryo was counted under a confocal microscope in embryos stained with DAPI, *n* = 15–20. Results are presented as the mean ± SEM. ***P < 0.001

And then we examined the activation of caspase-3 which plays important roles in apoptosis (Kidd, 1998; Porter and Janicke, 1999) by cleaved caspase-3 immunofluorescence staining to understand how Noc4l deficiency results in blastocyst failure. Compared with wild-type or heterozygous morulae in which few signals were detectable, activated caspase-3 was present in the Noc4l^−/−^ morula embryos (Fig. 2C). DAPI staining also indicated that fragmentation of chromatin had occurred, which is a known specific feature of apoptosis, in the Noc4l^−/−^ morulae (Fig. 2C). Additionally, the number of nuclei was significantly decreased in the Noc4l-deficient morulae compared with the wild-type or heterozygous embryos (Fig. 2D and 2E). These results indicated that the loss of functional Noc4l increases apoptosis and inhibits proliferation at the morula stage which results in deficient embryonic growth.

In the present study, we evaluated the physiological function of the mammalian NOC4L in vivo and generated the first Noc4l-deficient mouse model using the Cre-loxP approach. Our data reveal that the lack of functional Noc4l results in embryonic lethality during preimplantation stages of development. These results provide the first in vivo genetic evidence that Noc4l plays important roles in early embryogenesis in mice.

## Electronic supplementary material

Below is the link to the electronic supplementary material.

Supplementary material 1 (PDF 152 kb)

Supplementary material 2 (PDF 162 kb)

Supplementary material 3 (PDF 30 kb)
