# Supplementary material for: Targeted disruption of Noc4l leads to preimplantation embryonic lethality in mice
Source: Protein Cell. 2016 Dec 24;8(3):230–5. doi: 10.1007/s13238-016-0335-9 (PMC5326621; doi:10.1007/s13238-016-0335-9)
Supplement: Supplementary file 1 — Supplementary material 1 (PDF 152 kb) [file 13238_2016_335_MOESM1_ESM.pdf]

Materials and Methods

Mice

Eight-to-12-week-old female and male C57BL/6 mice were obtained from the Vitalriver (China) and housed in specific pathogen–free conditions. All experiments were conducted according to the guiding principles for the care and use of laboratory animals and were approved by the ethics committee of China Agricultural University (the reference number SKLAB-2014-01-15).

Construction of a phylogenetic tree of NOC4L Gene

Protein sequences were downloaded from UniprotKB (http://www.uniprot.org/) using the following accession numbers: Homo sapiens (Human), Q9BVI4; Mus musculus (Mouse), Q3T9T2; Pan troglodytes (Chimpanzee), K7BST0; Macaca mulatta (Rhesus macaque), F7HF96; Canis lupus familiaris (Dog), F1Q279; Rattus norvegicus (Rat), Q5I0I8; Gallus gallus (Chicken), Q5ZJC7; Saccharomyces cerevisiae (strain ATCC 204508 / S288c) (Baker's yeast), Q06512; Bos taurus (Bovine), F1MFW8; and Danio rerio (Zebrafish), F1R1T1. The phylogenetic relationship of the sequences was estimated by the neighbor-joining algorithm using MEGA6 software.

RNA extraction and quantitative RT-PCR analysis

C57BL/6 male mic were anaesthetized and tissues derived from heart, brain, liver, lung, kidney, small intestine, colon, muscle, epididymal fat (EpiWAT), brown adipose tissue (BAT), lymphoid organs (*i.e.*, spleen, thymus, lymphaden) and testes were collected and stored at -80°C until they were analyzed. Total RNA from mouse

tissues was extracted using Trizol reagent (Invitrogen, USA) according to the manufacturer’s instructions. cDNA was prepared from 1 μg of total RNA using M-MLV reverse transcriptase (Takara, Japan) according to the manufacturer’s instructions. Noc4l mRNA expression in mouse tissues was determined by quantitative RT-PCR (qPCR) using a Lightcycler 480 SYBR Green I Master Mix in a Lightcycler 480 real-time PCR machine (Roche, Germany). The cycling conditions were 95°C for 5 min, followed by 40 cycles at 95°C for 10 s, 60°C for 20 s and 72°C for 10 s. Gene expression levels were normalized to GAPDH (Baker et al., 2008; Baker et al., 2011; Liu et al., 2007). The fold-change in the mRNA expression levels was calculated using the comparative cycler method ($2^{-\Delta\Delta t}$). Primers sequences are listed as follows: GAPDH, forward, 5’-TGTGTCCGTCGTGGATCTGA-3’, and reverse, 5’-TTGCTGTTGAAGTCGCAGGAG-3’; Noc4l, forward, 5’-GAGGCAGTGCTGACGAGTC-3’, and reverse, 5’-GAGCCCACGAACAGCTCTTC-3’.

Isolation of RNA from different stages of preimplantation embryos

Total RNA was extracted from pooled preimplantation embryos using RNAprep Pure Micro kit (TIANGEN, China) according to the manufacturer’s instructions. C57BL/6 female mice were superovulated by an intraperitoneal injection of pregnant mare serum gonadotropin (PMSG, 10 IU/animal; Sigma, USA) and intraperitoneal injection of human chorionic Gonadotropin (hCG, 10 IU/animal; Sigma, USA) 48 h later. The mice were then allowed to mate with C57BL/6 male mice. The males were removed the next morning, and the females were examined for the presence of vaginal plugs, and this time point was designated E0.5. One-cell, 2-cell and morulae embryos

at E0.5, E1.5 and E2.5, respectively, were collected from the oviducts. E3.5 blastocysts were collected by flushing the uteri. Unfertilized eggs were collected without mating. Unfertilized eggs and 1-cell stage embryos were then treated with 0.5 mg ml^{-1} hyaluronidase solution (Sigma, USA) to remove the cumulus cells. At least 10 embryos each stage were pooled together. RNA from each stage was isolated after the addition of 20 ng carrier RNA and total RNA from each stage was subjected to reverse transcription for complementary DNA synthesis with SuperScript III RT using random hexamers according to the manufacturer's protocol (Invitrogen, USA). Noc4l mRNA expression at each stage of preimplantation embryos was determined by qPCR as mentioned above.

Immunofluorescence

Embryos were fixed in 4 % PFA in PBS for 30 min at room temperature. After washing three times with PBS containing 0.1 % BSA, embryos were permeabilized with 0.2 % Triton X-100 in PBS for 15 min at room temperature. Embryos were then incubated with the primary Noc4l antibody (diluted 1:200 in PBS, HPA046362, Sigma, USA) overnight at 4˚C in a humidified chamber. Embryos were washed three times with fresh PBS to remove excess primary antibody and then incubated with Alexa Fluor 594-conjugated goat anti-rabbit IgG secondary antibody (1:200 dilution, ZSGB-BIO, China) for 1 h at 37˚C. After nuclear DNA staining with 1 μg ml^{-1} 4', 6'-diamidino-2-phenylindole (DAPI) in PBS for 10 min at room temperature, the embryos were mounted on a glass slide and sealed with nail polish. Images were captured using confocal microscope (Olympus, Japan).

Generation and sub-cellular localization of the EGFP- and Flag-tagged NOC4L

The open reading frame (ORF) of NOC4L was amplified by PCR according to the cDNA of Hela cells (ATCC, USA) and then was subcloned into the pEGFP-C1 or pEGFP-N1 vector (Clontech, USA) in order to generate the EGFP-NOC4L and NOC4L-EGFP fusion protein, respectively. NOC4L tagged with Flag at the amino or carboxyl terminus was generated by PCR and subcloned into the expression plasmid pCDA3.1 (Invitrogen, USA). Hela cells were cultured in high-glucose Dulbecco’s modified Eagle’s medium (Sigma, USA) supplemented with 10% FBS (HyClone, USA) at 37°C in an incubator with 5% CO_2 and were seeded onto 24-well plates 1
(corning, USA) one day before transfection. Next day, the recombinant plasmids and empty vectors, respectively were transfected using lipofectamine 3000 (Invitrogen, USA) according to the manufacturer’s instructions. After 24h transfection, the EGFP signals were detected by fluorescence microscopy and the nuclear DNA was stained by DAPI. Immunofluorescence assay was used to detect Flag-tagged NOC4L by using anti-Flag antibody (Santa Cruz, USA) followed by FITC-conjugated secondary antibody. All primers used for constructs are available upon request.

Construction of a targeting vector and generation of Noc4l mutant mice

Mice with floxed Noc4l alleles were generated by the Model Animal Research Center of Nanjing University in China. Briefly, the Noc4l-conditional knock out (KO) mice ($Noc4l^{flox/flox}$) was constructed to flank exon 3 of the mouse Noc4l gene with two loxP sites as described previously (Fig. S2A)(Xia et al., 2013). The vector was transfected into embryonic stem (ES) cells from Sv129 mice. After neomycin selection, the ES clones flanking loxP sites were microinjected into C57BL/6 mouse blastocysts. Heterozygous $Noc4l^{+/flox}$ mice were obtained after several rounds of

selection, and Noc4l$^{flox/flox}$ mice were generated by intercrossing Noc4l$^{+/flox}$ mice. Noc4l$^{flox/flox}$ mice were bred with EIIa-Cre transgenic mice (Lakso et al., 1996) to facilitate the deletion of exon 3 and produce Noc4l$^{+/-}$ mice. Noc4l$^{-/-}$ mice were generated by intercrossing Noc4l$^{+/-}$ mice.

PCR Genotyping

Weaned mice derived from heterozygous intercrosses were screened for the targeted disruption of Noc4l using PCR. Mouse tail clippings were collected and heated with 75 µL of alkaline lysis reagent (25 mM NaOH and 0.2 mM disodium EDTA, pH 12) at 98°C for 1 h (Truett et al., 2000). Genomic DNA was extracted after neutralization using 75 µL of neutralizing reagent (40 mM Tris-HCl pH 5). Two microliters of the extracted DNA was used in a standard 20-µL PCR reaction with rTaq polymerase (Takara, Japan). The following primers were used: loxp-F 5'-GCCTTGTCATAGACCATGCGATCTG-3' and loxp-R 5'-TAAGATGCCAGACCGGGGCTTG-3' for the loxP site generating the 384-bp (WT) and 502-bp (flox/flox) alleles, respectively. The cycling parameters were 95°C for 5 min, followed by 30 cycles at 95°C for 30 s, 56°C for 30 s and 72°C for 30 s, and a final extension at 72°C for 10 min. In experiments using the primers loxp-F1 5'-GCCATTTCCGAGTTTGATACTGTCT-3' and Cko-R 5'-CTAACTATTCCCTTGTCCCCCCCA-3' to identify deficient genotypes generating 700-bp (WT) and 184-bp (KO) fragments, the cycling parameters were 95°C for 5 min, followed by 35 cycles at 95°C for 30 s, 62.3°C for 30 s and 72°C for 90 s, and a final extension at 72°C for 10 min. Ten microliters of each reaction mixture were separated on a 2.0 % agarose gel in 1X Tris acetate-EDTA buffer.

Timed pregnancies

To generate the timed pregnancies, $Noc4l^{+/-}$ females were superovulated and then were mated with $Noc4l^{+/-}$ males as described above. Plugged females were sacrificed at E1.5, 2.5, 3.5, and E8.5 to E16.5 to isolate the embryos, which were then genotyped by PCR as described below.

Embryo genotyping

Embryo genotyping was performed on E8.5 to E16.5 by PCR analysis. Dissected embryo tissues were washed in PBS and digested overnight in 500 μL of lysis buffer (100 mM Tris-HCl pH 8.5, 5 mM EDTA, 0.2 % SDS, 200 mM NaCl and 50 μg of proteinase K) at 55°C. After digestion, the DNA was purified by ethanol precipitation and used for PCR genotyping as described above.

Nested PCR was used to genotype preimplantation embryos (E1.5, E2.5, and E3.5) derived from the heterozygous intercrosses. Each preimplantation embryo collected from timed pregnant females was lysed for 4 h at 55°C in embryonic lysis buffer (50 mM KCl, 10 mM Tris-HCl PH 8.3, 2 mM $MgCl_2$, 0.1 mg ml^{-1} galectin, 0.45 % NP40, 0.45 % Tween-20 and 500 μg ml^{-1} proteinase K), and proteinase K was subsequently inactivated at 95°C for 10 min. The first round of nested PCR was carried out using embryonic DNA with the primers F1 5'-TTGCCAGGACTGCGTGAA-3' and R1 5'-GCAGGGGAGGCCACCTAACTATTC-3' in a total volume of 20 μL. Next, 2 μL of the first round PCR product was used for the second round of PCR amplification for 30 cycles with the primers loxp-F1 and Cko-R as described above.

In vitro cultures of the preimplantation embryos

To observe the development of the preimplantation embryos in vitro, embryos at the 2-cell stage were collected by flushing the oviduct of plugged females with M2 medium (Millipore, USA) 40–42 h after injection of hCG. After washing three times with M2 medium, the embryos were placed into 96-well plates and individually cultured in a 50 μL KSOM medium (Millipore, USA) under mineral oil in a 5 % CO_2 environment at 37°C. The growth patterns of the embryos were examined and imaged by microscopy (Olympus, Japan) at 6 h intervals.

Apoptosis assay

E2.5 embryos were harvested after Noc4l$^{+/-}$ intercrossed and the embryos were cultured in KSOM medium for 12 h under the mineral oil in a 5 % CO_2 environment at 37°C. Embryos were then fixed and permeabilized as described above. After washing three times with PBS, embryos were incubated with the primary cleaved caspase-3 antibody (diluted 1:200 in PBS, 9664s; Cell Signaling Technology, USA) overnight at 4°C in a humidified chamber. Embryos were washed three times with fresh PBS to remove excess primary antibody and then incubated with Alexa Fluor 594-conjugated goat anti-rabbit IgG secondary antibody (1:200, ZSGB-BIO, China) for 1 h at 37°C. After nuclear DNA staining with DAPI in PBS for 10 min at room temperature, the embryos were mounted on a glass slide. Images were captured using confocal microscope (Olympus, Japan). In addition, the genotypes of the embryos evaluated were determined by the methods as described above.

References

Baker, D.J., Perez-Terzic, C., Jin, F., Pitel, K.S., Niederlander, N.J., Jeganathan, K., Yamada, S., Reyes, S., Rowe, L., Hiddinga, H.J., *et al.* (2008). Opposing roles for p16Ink4a and p19Arf in senescence and ageing caused by BubR1 insufficiency. Nature cell biology *10*, 825-836.

Baker, D.J., Wijshake, T., Tchkonia, T., LeBrasseur, N.K., Childs, B.G., van de Sluis, B., Kirkland, J.L., and van Deursen, J.M. (2011). Clearance of p16Ink4a-positive senescent cells delays ageing-associated disorders. Nature *479*, 232-236.

Lakso, M., Pichel, J.G., Gorman, J.R., Sauer, B., Okamoto, Y., Lee, E., Alt, F.W., and Westphal, H. (1996). Efficient in vivo manipulation of mouse genomic sequences at the zygote stage. Proc Natl Acad Sci U S A *93*, 5860-5865.

Liu, C.L., Yu, I.S., Pan, H.W., Lin, S.W., and Hsu, H.C. (2007). L2dtl is essential for cell survival and nuclear division in early mouse embryonic development. J Biol Chem *282*, 1109-1118.

Truett, G.E., Heeger, P., Mynatt, R.L., Truett, A.A., Walker, J.A., and Warman, M.L. (2000). Preparation of PCR-quality mouse genomic DNA with hot sodium hydroxide and tris (HotSHOT). Biotechniques *29*, 52, 54.

Xia, P., Wang, S., Du, Y., Zhao, Z., Shi, L., Sun, L., Huang, G., Ye, B., Li, C., Dai, Z., *et al.* (2013). WASH inhibits autophagy through suppression of Beclin 1 ubiquitination. EMBO J *32*, 2685-2696.
