# Supplementary material for: Targeted disruption of Noc4l leads to preimplantation embryonic lethality in mice
Source: Protein Cell. 2016 Dec 24;8(3):230–5. doi: 10.1007/s13238-016-0335-9 (PMC5326621; doi:10.1007/s13238-016-0335-9)
Supplement: Supplementary file 2 — Supplementary material 2 (PDF 162 kb) [file 13238_2016_335_MOESM2_ESM.pdf]

Fig. S1

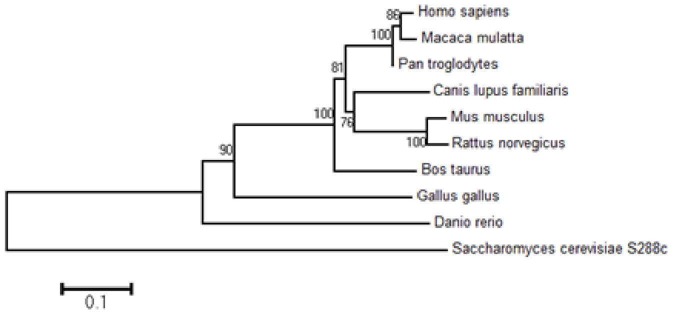

Fig. S1 The conservation of the NOC4L protein among different species

Phylogenetic relationships of NOC4L proteins. The protein sequences used are provided in the material and methods section. The phylogenetic relationship of the sequences was estimated using the neighbor-joining algorithm with MEGA6 software.

Branch lengths are proportional to the amount of evolution predicted to occur with each lineage. Numbers refer to the percentage of bootstrap replications that support each node.

Fig. S2

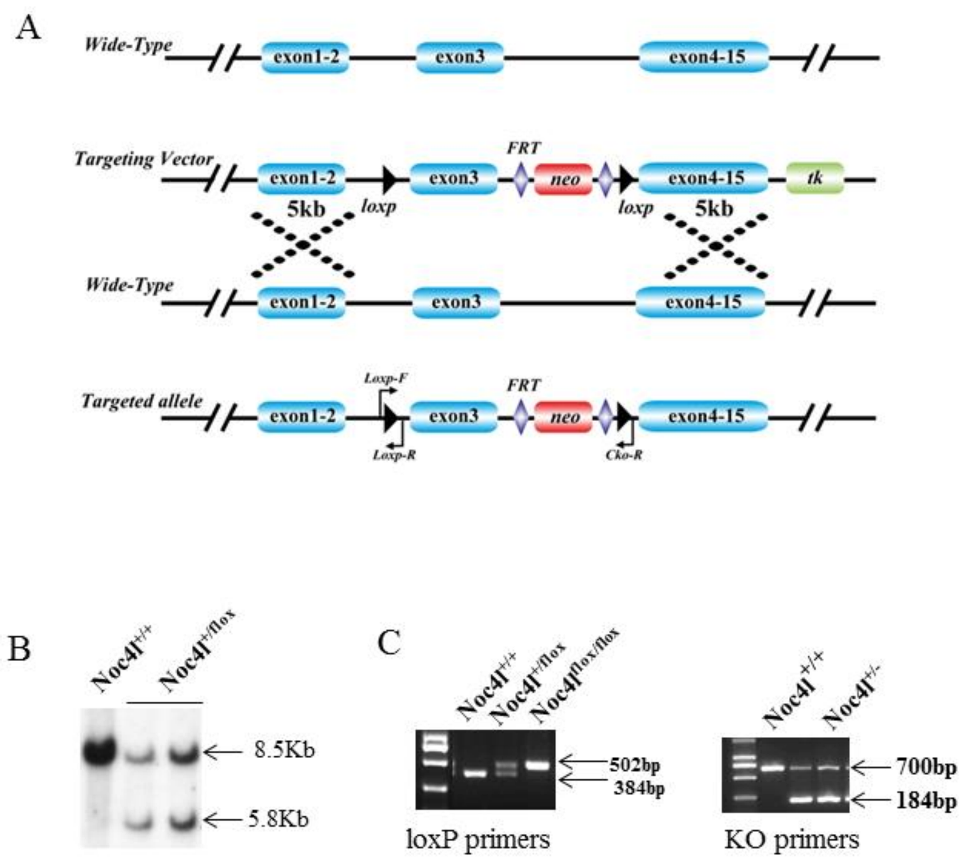

Fig. S2 Generation of Noc4l-knockout (Noc4l$^{-/-}$) mice.

(A) Schematic representation of the mouse Nocc4l genomic locus and the targeting strategy used to generate the conditional knockout of the Noc4l gene by deleting exon 3. The coding exons of the Noc4l gene are represented by blue boxes. The target vector with exon 3 of the Noc4l gene was flanked by two loxP sites (triangle) and with one neomycin resistance gene. The primers used were loxP primers (loxp-F/loxp-R) for loxP site identification and KO primers (loxp-F1/Cko-R) for deficient genotypes. (B) Southern blot analysis. Genomic DNA was isolated from targeted ES cells and digested with BamHI. Digestion of wild-type allele (Noc4l$^{+/+}$) yields an 8.5-Kb fragment, while the digestion of the mutant allele yields a 5.8-Kb fragment. (C) Genotyping of Noc4l$^{+/+}$ and Noc4l$^{+/-}$ mice by PCR analysis. The DNA template was extracted from mouse tails. The loxP site was identified using loxP primers (384bp/502bp, right panel). The genotypes of the offspring were analyzed by PCR using the KO primers (184bp/700bp, left panel).
