# Supplementary material for: Targeted disruption of Noc4l leads to preimplantation embryonic lethality in mice
Source: Protein Cell. 2016 Dec 24;8(3):230–5. doi: 10.1007/s13238-016-0335-9 (PMC5326621; doi:10.1007/s13238-016-0335-9)
Supplement: Supplementary file 3 — Supplementary material 3 (PDF 30 kb) [file 13238_2016_335_MOESM3_ESM.pdf]

**Table S1 Genotype analysis of offspring from Noc4l$^{+/-}$ intercrosses**

| Stage | Number analyzed | Genotype | | | Resorption |
|---|---|---|---|---|---|
| | | +/+ | +/- | -/- | |
| Weaning | 204 | 66(32.4%)[a] | 138(66.7%) | 0 | NA |
| E11.5-16.5 | 63 | 24(38.1%) | 39(61.9%) | 0 | 1 |
| E8.5-10.5 | 57 | 19(33.3%) | 38(66.7%) | 0 | 8 |
| E4.5 | 55 | 11(20%) | 33(60%) | 11(20%) | NA |
| 2-Cell | 114 | 32(28.1%) | 57(50%) | 25(21.9%) | NA |

NA, not applicable.

[a]The proportion of each genotype from Noc4l$^{+/-}$ intercrosses are shown.
